# Synthesis, crystal structure and Hirshfeld surface analysis of (3*Z*)-4-[(4-amino-1,2,5-oxa­diazol-3-yl)amino]-3-bromo-1,1,1-tri­fluoro­but-3-en-2-one

**DOI:** 10.1107/S2056989024004080

**Published:** 2024-05-10

**Authors:** Firudin I. Guseinov, Sevim Türktekin Çelikesir, Mehmet Akkurt, Viacheslav O. Ovsyannikov, Bogdan I. Ugrak, Oksana M. Lavrova, Aida I. Samigullina, Ajaya Bhattarai

**Affiliations:** a Kosygin State University of Russia, 117997 Moscow, Russian Federation; b N. D. Zelinsky Institute of Organic Chemistry, Russian Academy of Sciences, 119991 Moscow, Russian Federation; c Department of Physics, Faculty of Sciences, Erciyes University, 38039 Kayseri, Türkiye; d MIREA, Russian Technology University, Lomonosov Institute of Fine Chemical Technology, Moscow, 119571, Russian Federation; e Department of Chemistry, M.M.A.M.C (Tribhuvan University), Biratnagar, Nepal; University of Neuchâtel, Switzerland

**Keywords:** crystal structure, α-haloketone, di­amino­furazan, non-covalent inter­actions, oxa­diazole ring, disorder, Hirshfeld surface analysis

## Abstract

In the title compound, C_6_H_4_BrF_3_N_4_O_2_, the oxa­diazole ring is essentially planar with a maximum deviation of 0.003 (2) Å. In the crystal, mol­ecular pairs are connected by N—H⋯N hydrogen bonds, forming dimers with an *R* ^2^_2_(8) motif. The dimers are linked into layers parallel to the (10  ) plane by N—H⋯O hydrogen bonds. In addition, C—O⋯π and C—Br⋯π inter­actions connect the mol­ecules, forming a three-dimensional network. The F atoms of the tri­fluoro­methyl group are disordered over two sites in a 0.515 (6): 0.485 (6) ratio. The inter­molecular inter­actions in the crystal structure were investigated and qu­anti­fied using Hirshfeld surface analysis.

## Abstract 2

In the crystal, mol­ecular pairs are connected by N—H⋯N hydrogen bonds, forming dimers with an 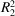 (8) motif. The dimers are linked into layers parallel to the (10 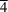 ) plane by N—H⋯O hydrogen bonds. In addition, C—O⋯π and C—Br⋯π inter­actions connect the mol­ecules, forming a three-dimensional network.

## 1. Chemical context

Among the main trends in the development of organic chemistry over the past 20 years, one can note the key role and rapid development of the chemistry of organofluorine compounds (Meanwell, 2018 ▸). This is due to the extremely high practical importance of organofluorine mol­ecules. The introduction of fluorine into the target mol­ecule changes such important parameters as lipophilicity, solubility, binding to receptors, metabolism, acid–base characteristics, and conformational properties of compounds. Currently, about 25% of new drugs and 35% of substances used in agriculture (agrochemicals) contain at least one fluorine atom (Chandra *et al.*, 2023 ▸; Han *et al.*, 2020 ▸; Mei *et al.*, 2019 ▸; Shabir *et al.*, 2023 ▸; Zhang *et al.*,, 2022 ▸).

Di­amino­furaza­nes and their derivatives are widely used to obtain useful heterocyclic compounds, high-energy explosives with great potential application value, anti­microbials, highly effective biocidal and anti­tumor agents, as well as in photochemistry (Chang *et al.*, 2023 ▸; Chen *et al.*, 2022 ▸; Dutta *et al.*, 2022 ▸; Liao *et al.*, 2020 ▸; Liu *et al.*, 2022 ▸; Ugrak *et al.*, 2023 ▸). Similarly to other *N*-ligands (Gurbanov *et al.*, 2022*a* ▸,*b* ▸; Kopylovich *et al.*, 2011*a* ▸,*b* ▸, 2012 ▸), new derivatives of furazan can also be used in crystal engineering (Gurbanov *et al.*, 2020 ▸) as well as the synthesis of coordination compounds for catal­ysis (Mac Leod *et al.*, 2012 ▸; Mahmudov *et al.*, 2013 ▸; Mizar *et al.*, 2012 ▸) and biological studies (Martins *et al.*, 2017 ▸). In fact, the non-covalent bond-acceptor ability of the furazan motif can be employed as a unique tool for crystal engineering. We believe that the combination of tri­fluoro­methyl and furazan fragments in one mol­ecule can lead to the synthesis of new compounds with useful properties. Therefore, we studied the condensation of (*Z*)-3-bromo-4-eth­oxy-1,1,1-tri­fluoro­but-3-en-2-one with di­amino­furazan in different polar solvents, with the best yield being in ethanol. It was shown that the reaction occurs only with the participation of the vinyl fragment and the active ketone group is not affected. The condensation product is an enamine, and its structure was confirmed by NMR spectros­copy and X-ray diffraction analysis. 
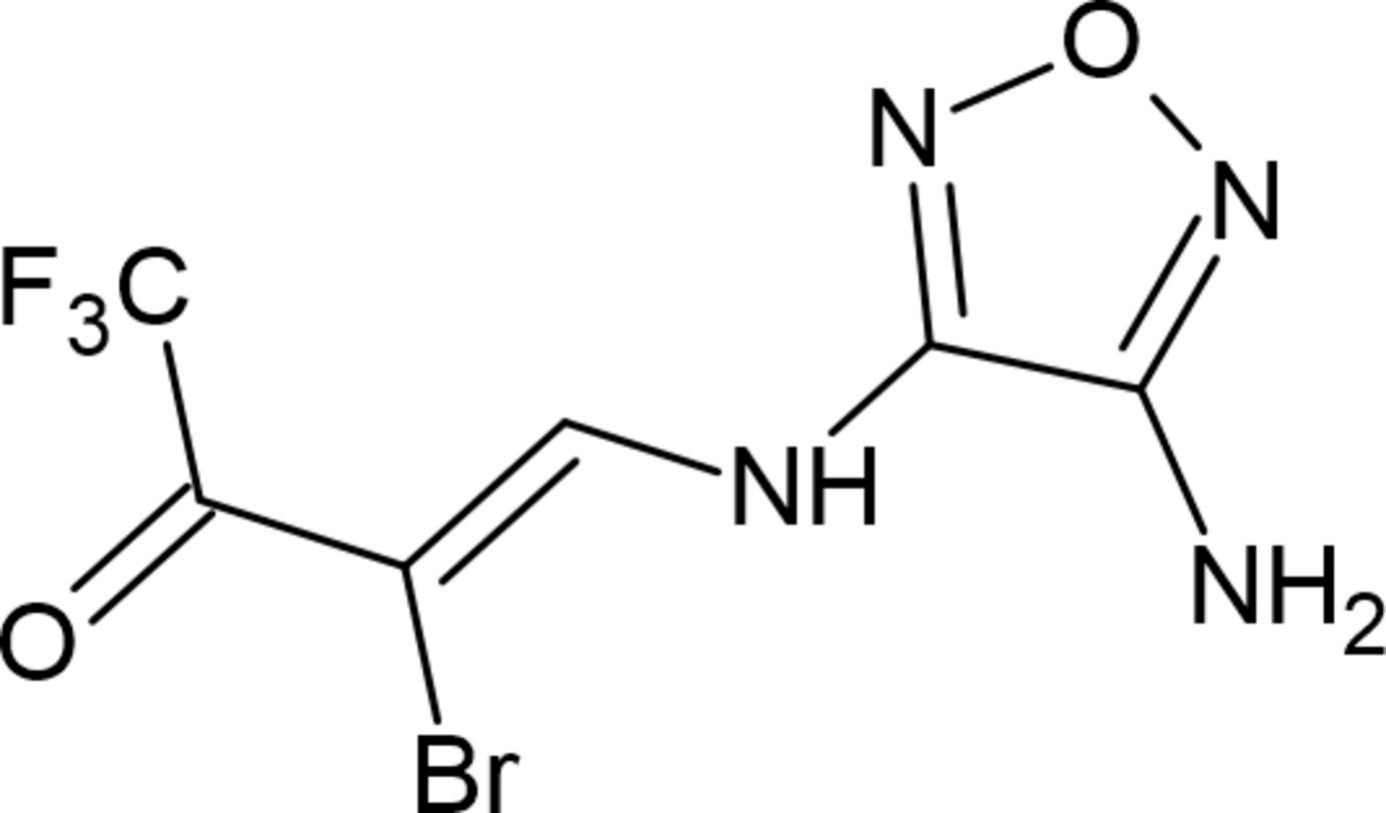


## 2. Structural commentary

**Figure 1:**
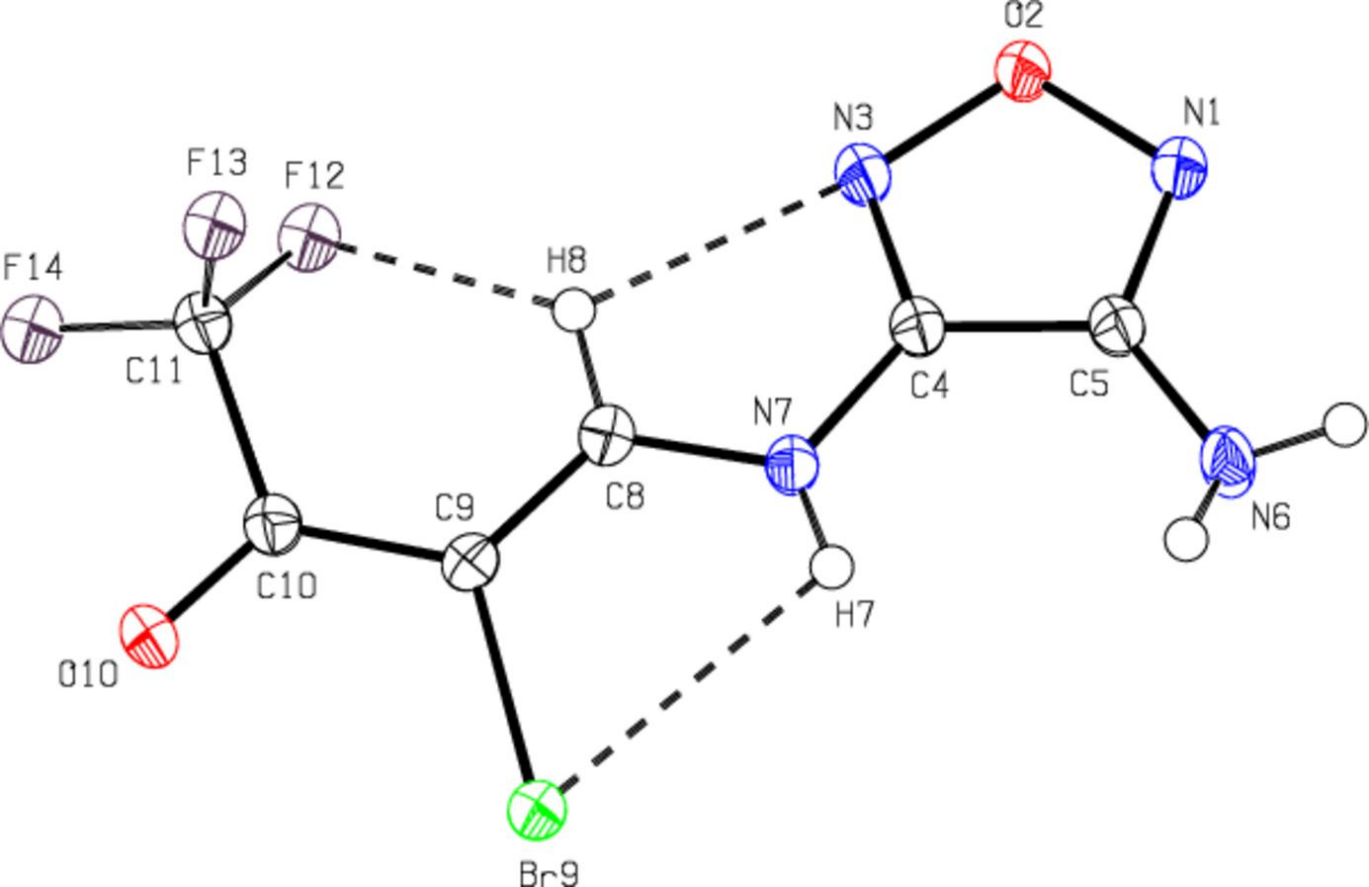
Mol­ecular structure of the title compound, showing the atom-labeling scheme and with displacement ellipsoids drawn at the 50% probability level. The intra­molecular N—H⋯Br, C—H⋯F and C—H⋯N hydrogen bonds are shown as dashed lines. Only the major disorder component is shown for clarity.

In the title compound (Fig. 1 ▸), the oxa­diazole ring (N1/O2/N3/C4/C5) is essentially planar [maximum deviation = 0.003 (2) Å for C4]. In the mol­ecule, the intra­molecular N—H⋯Br, C—H⋯F and C—H⋯N hydrogen bonds form *S*(5), *S*(6) and *S*(5) ring motifs, respectively (Bernstein *et al.*, 1995 ▸; Table 1 ▸; Fig. 1 ▸). The N3—C4—N7—C8, C4—N7—C8—C9, C8—C9—C10—O10 and C8—C9—C10—C11 torsion angles are −7.5 (4), 173.0 (2), −178.3 (2) and −2.8 (4)°, respectively. The geometric parameters are normal and comparable to those of related compounds listed in the *Database survey* section.

## 3. Supra­molecular features and Hirshfeld surface analysis

**Figure 2:**
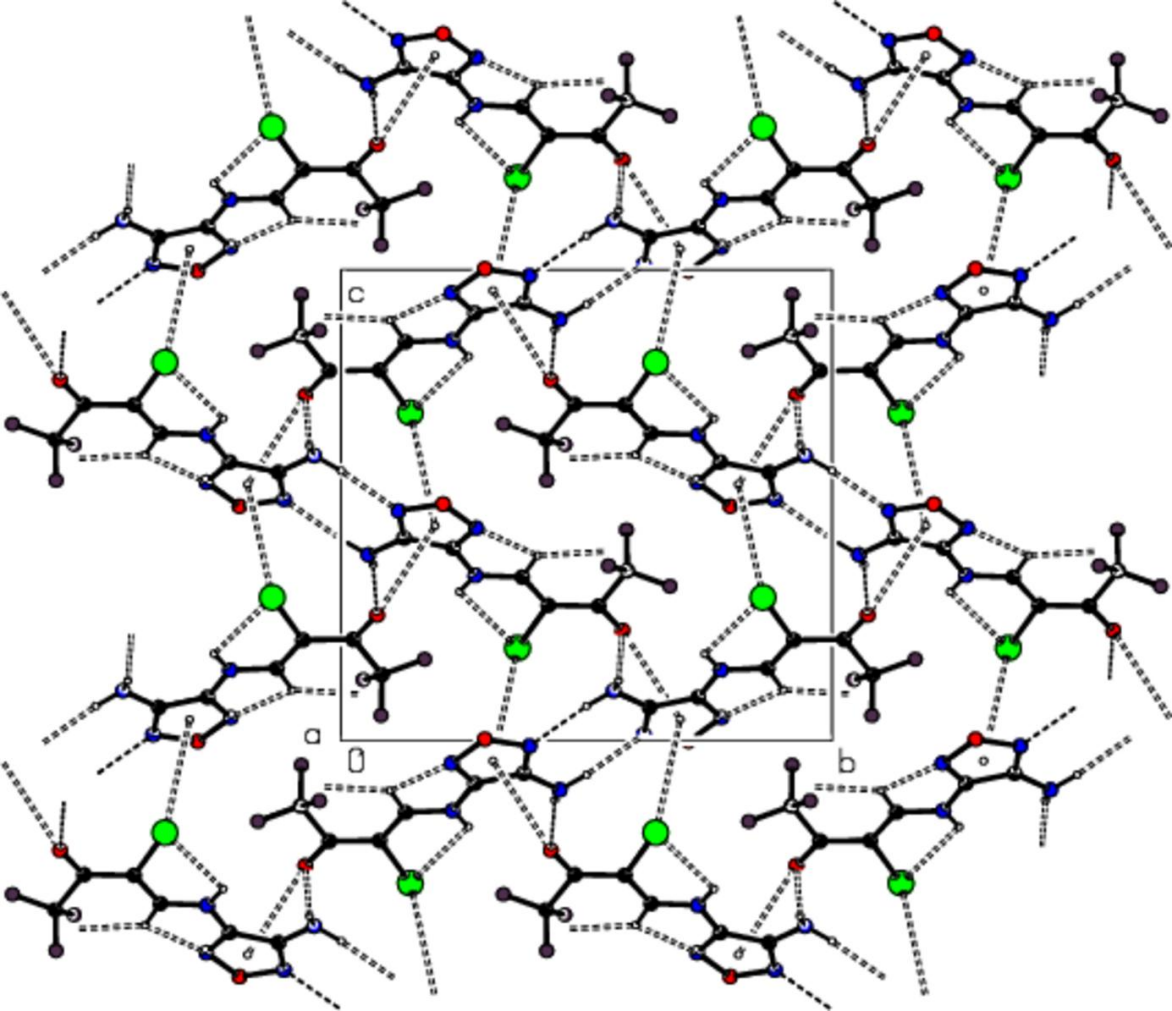
Views of the intra­molecular N—H⋯Br, C—H⋯F, C—H⋯N hydrogen bonds, the inter­molecular N—H⋯O, N—H⋯N hydrogen bonds, and the C—O⋯π and C—Br⋯π inter­actions along the *a*-axis.

In the crystal, mol­ecular pairs are connected by N—H⋯N hydrogen bonds, forming dimers with an 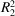 (8) motif (Bernstein *et al.*, 1995 ▸; Table 1 ▸; Fig. 2 ▸). The dimers are linked into layers parallel to the (10 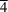 ) plane by N—H⋯O hydrogen bonds (Table 1 ▸; Fig. 2 ▸). In addition, C—O⋯π and C—Br⋯π inter­actions connect the mol­ecules, forming a three-dimensional network (Figs. 2 ▸ and 3 ▸).

**Figure 4:**
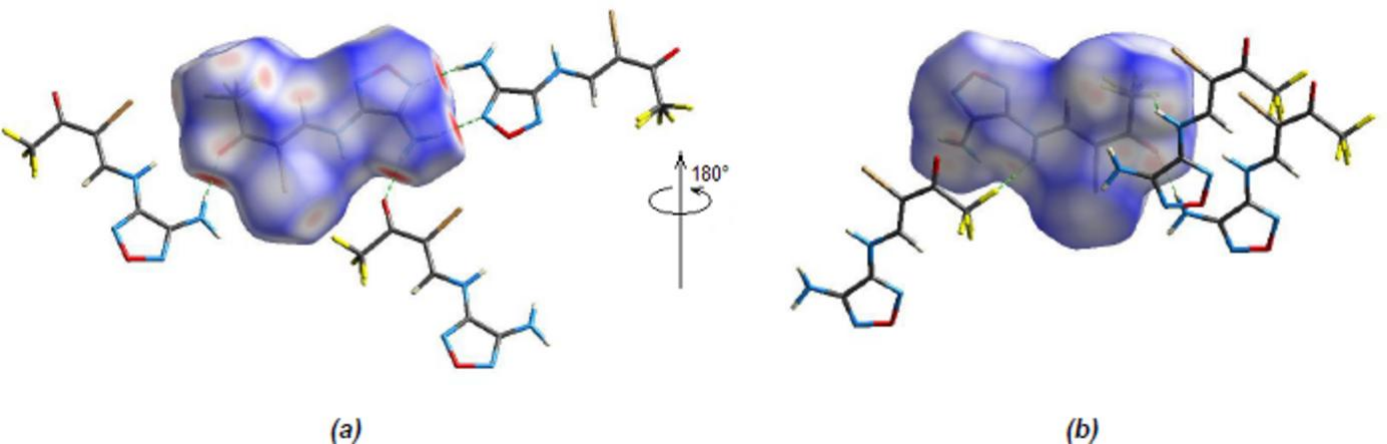
The three-dimensional Hirshfeld surface for the title compound, plotted over *d*_norm_.

To qu­antify the inter­molecular inter­actions, a Hirshfeld surface analysis was performed and *CrystalExplorer17.5* (Spackman *et al.*, 2021 ▸) was used to obtain two-dimensional fingerprint plots. Fig. 4 ▸ shows the Hirshfeld surface mapped over *d*_norm_ using a common surface resolution and a constant color scale of −0.5339 (red) to + 0.9642 (blue) a.u. On the Hirshfeld surface, shorter and longer contacts are indicated by red and blue spots, respectively, and contacts with lengths about equal to the sum of the van der Waals radii are indicated by white spots.

**Figure 5:**
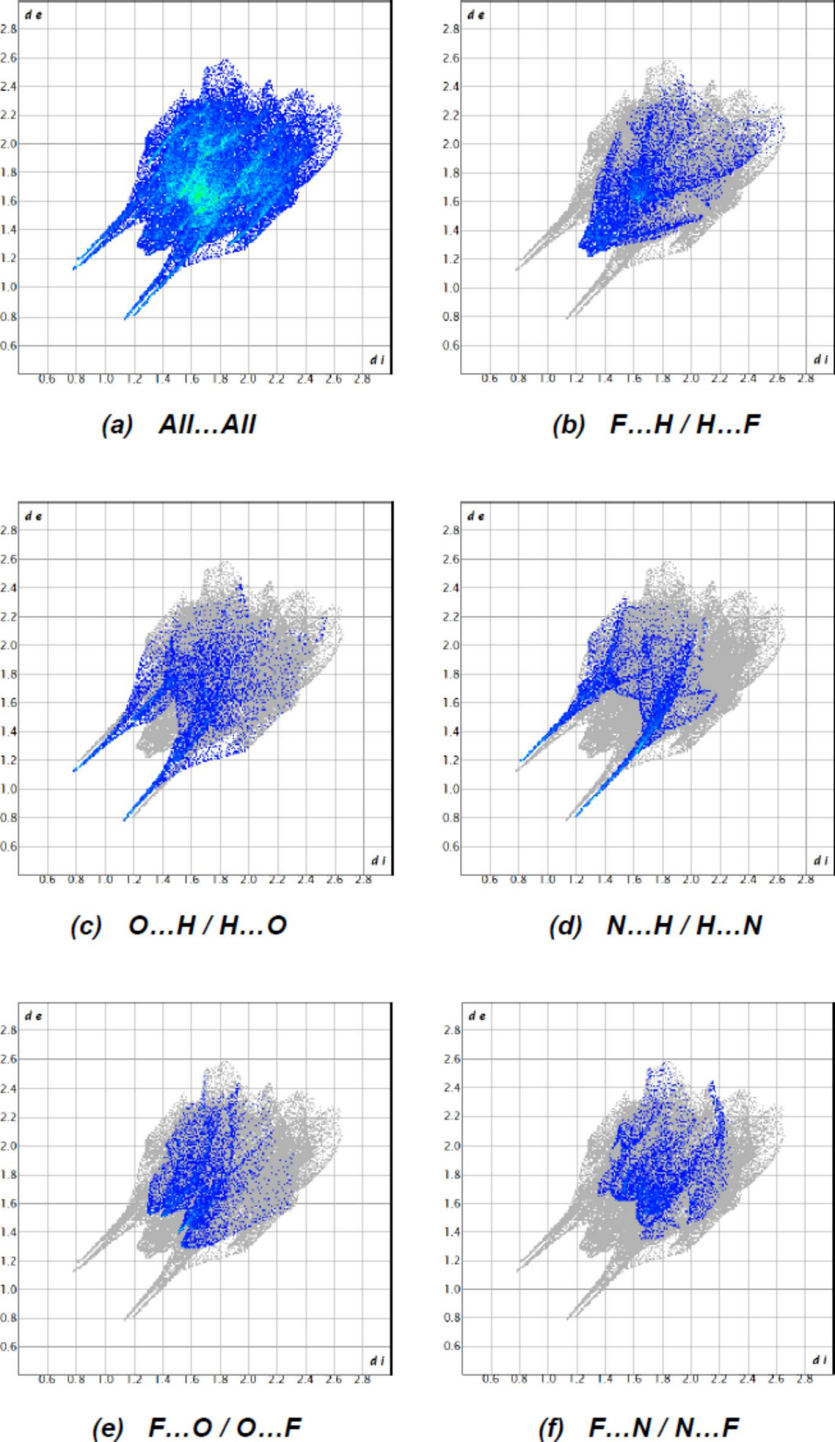
A view of the two-dimensional fingerprint plots for the title compound, showing (*a*) all inter­actions, and delineated into (*b*) F⋯H/H⋯F, (*c*) O⋯H/H⋯O, (*d*) N⋯H/H⋯N, (*e*) F⋯O/O⋯F, and (*f*) F⋯N/N⋯F inter­actions. The *d*_i_ and *d*_e_ values are the closest inter­nal and external distances (in Å) from given points on the Hirshfeld surface.

Fig. 5 ▸ depicts the two-dimensional fingerprint plots of (*d*_i_, *d*_e_) points from all contacts contributing to the Hirshfeld surface analysis in normal mode for all atoms. The most important inter­molecular inter­actions are F⋯H/H⋯F, O⋯H/H⋯O and N⋯H/H⋯N contacts, contributing to 12.8%, 11.9% and 10.7%, respectively, to the overall crystal packing. Other inter­actions and their respective contributions are F⋯O/O⋯F (8.8%), F⋯N/N⋯F (7.4%), F⋯F (6.3%), Br⋯H/H⋯Br (5.7%), Br⋯F/F⋯Br (5.2%), F⋯C/C⋯F (4.9%), Br⋯C/C⋯Br (4.5%), Br⋯O/O⋯Br (3.8%), Br⋯N/N⋯Br (3.5%), O⋯C/C⋯O (3.4%), N⋯C/C⋯N (3.1%), C⋯H/H⋯C (3.0%), O⋯N/N⋯O (2.3%), H⋯H (2.2%) and N⋯N (0.4%).

## 4. Database survey

A search of the Cambridge Structural Database (CSD, Version 5.42, update of September 2021; Groom *et al.*, 2016 ▸) gave four compounds closely related to the title compounds, *viz*. CSD refcodes KIMZEP (**I**: Okmanov *et al.*, 2023 ▸), KIMZIT (**II**: Okmanov *et al.*, 2023 ▸), ZARJEJ (**III**: Jia *et al.*, 2012 ▸) and PUHDUS (**IV**: Zhang *et al.*, 2009 ▸).

In the crystals of **I** and **II**, C—H⋯π inter­actions are observed between neighboring mol­ecules. In the crystal of **III**, one of the amino H atoms forms an intra­molecular N—H⋯N hydrogen bond; adjacent mol­ecules are linked by N—H⋯N hydrogen bonds, forming a chain running along [10 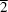 ]. In the crystal of **IV**, inter­molecular N—H⋯N, N—H⋯O, O—H⋯N and O—H⋯O hydrogen bonds link the mol­ecules into a three-dimensional network.

## 5. Synthesis and crystallization

Equimolar amounts of (*Z*)-3-bromo-4-eth­oxy-1,1,1-tri­fluoro­but-3-en-2-one (0.247 g, 1.0 mmol) and di­amino­furazan (0.100 g, 1.0 mmol) were dissolved in 25 ml of ethanol and refluxed for 3 h. The reaction was monitored by ^1^H NMR. A characteristic disappearance of the signals associated with the eth­oxy group was observed. At the end of the reaction, the solvent was removed *in vacuo*. (3*Z*)-4-[(4-amino-1,2,5-oxa­diazol-3-yl)amino]-3-bromo-1,1,1-tri­fluoro­but-3-en-2-one in the form of a yellow precipitate, which was then recrystallized from acetone. Yield 0.249 g (83%); m.p. 444–445 K. Analysis calculated (%) for C_6_H_4_BrF_3_N_4_O_2_: C 23.94, H 1.34, Br 26.54, F 18.93, N 18.93, O 10.63; found: C23.92, H 1.31, Br 26.57, F 18.93, N 18.94, O 10.60. ^1^H NMR (300 MHz, acetone-*d*_6_): 5.88 (*br*, 2H, NH_2_), 8.52 (*s*, 1H, CH), 9.05 (*br*, 1H, NH). ^13^C NMR (75 MHz, DMSO-*d*_6_): 97.89, 114.30, 120.06, 146.71, 147.01, 206.20. ESI–MS: *m*/*z*: 298.9408 [*M* − H]^+^.

## 6. Refinement

Crystal data, data collection and structure refinement details are summarized in Table 2 ▸. All H atoms were located in a difference map and freely refined. The F atoms of the tri­fluoro­methyl group are disordered over two sites in a 0.515 (6): 0.485 (6) ratio. The C—F bond lengths in the disordered tri­fluoro­methyl group were constrained to be the same (using SADI), as were the thermal parameters of the F atoms (using EADP).

## Supplementary Material

Crystal structure: contains datablock(s) I. DOI: 10.1107/S2056989024004080/tx2085sup1.cif

Structure factors: contains datablock(s) I. DOI: 10.1107/S2056989024004080/tx2085Isup2.hkl

Supporting information file. DOI: 10.1107/S2056989024004080/tx2085Isup3.cml

CCDC reference: 2352897

Additional supporting information:  crystallographic information; 3D view; checkCIF report

## Figures and Tables

**Figure 3:**
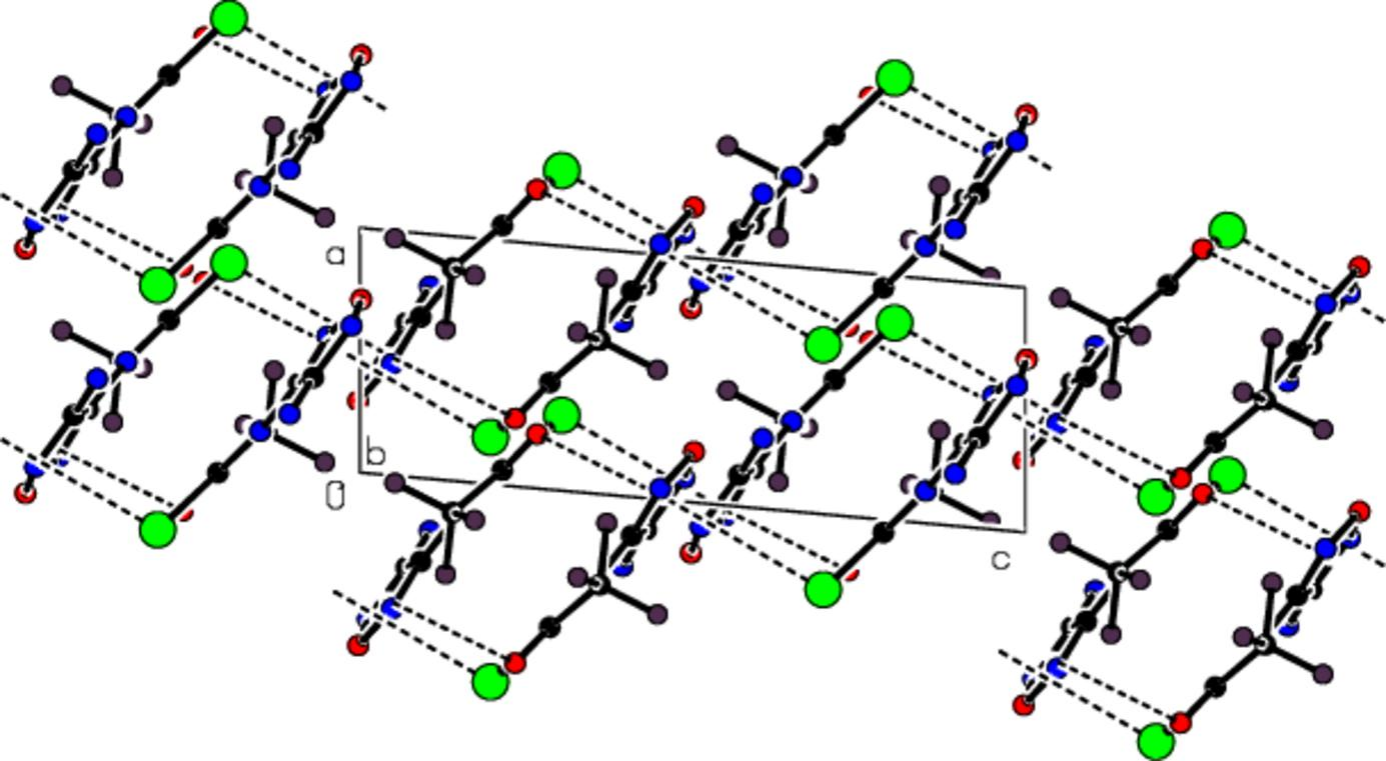
Packing viewed along the *b*-axis with the C—O⋯π and C—Br⋯π inter­actions indicated by dashed lines.

**Table 1:** Hydrogen-bond geometry (Å, °)

*D*—H⋯*A*	*D*—H	H⋯*A*	*D*⋯*A*	*D*—H⋯*A*
N6—H6*A*⋯O10^i^	0.77 (4)	2.14 (4)	2.871 (3)	159 (4)
N6—H6*B*⋯N1^ii^	0.90 (4)	2.11 (4)	2.995 (3)	169 (3)
N7—H7⋯Br9	0.81 (3)	2.76 (3)	3.127 (2)	109 (2)
C8—H8⋯F12	0.95 (3)	2.17 (3)	2.799 (5)	123 (2)
C8—H8⋯F12*A*	0.95 (3)	2.11 (3)	2.808 (5)	129 (2)
C8—H8⋯N3	0.95 (3)	2.38 (3)	2.759 (3)	103.3 (19)

Symmetry codes: (i) 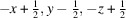 ; (ii) 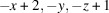 .

**Table 2:** Experimental details

Crystal data	
Chemical formula	C_6_H_4_BrF_3_N_4_O_2_
*M*_r_	301.04
Crystal system, space group	Monoclinic, *P*2_1_/*n*
Temperature (K)	100
*a*, *b*, *c* (Å)	4.95281 (3), 14.0515 (1), 13.5208 (1)
β (°)	95.1372 (6)
*V* (Å^3^)	937.19 (1)
*Z*	4
Radiation type	Cu *K*α
μ (mm^−1^)	6.46
Crystal size (mm)	0.21 × 0.04 × 0.04

Data collection	
Diffractometer	XtaLAB Synergy, Dualflex, HyPix
Absorption correction	Gaussian (*CrysAlis PRO*; Rigaku OD, 2023 ▸)
*T*_min_, *T*_max_	0.490, 1.000
No. of measured, independent and observed [*I* > 2σ(*I*)] reflections	24751, 2038, 2010
*R*_int_	0.030
(sin θ/λ)_max_ (Å^−1^)	0.638

Refinement	
*R*[*F* ^2^ > 2σ(*F* ^2^)], *wR*(*F* ^2^), *S*	0.028, 0.065, 1.09
No. of reflections	2038
No. of parameters	159
No. of restraints	3
H-atom treatment	All H-atom parameters refined
Δρ_max_, Δρ_min_ (e Å^−3^)	0.73, −0.50

Computer programs: *CrysAlis PRO* (Rigaku OD, 2023 ▸), *SHELXT* (Sheldrick, 2015*a* ▸), *SHELXL2018* (Sheldrick, 2015*b* ▸), *ORTEP-3 for Windows* (Farrugia, 2012 ▸) and *PLATON* (Spek, 2020 ▸).

## supplementary crystallographic information

### Crystal data

**Table:**

C_6_H_4_BrF_3_N_4_O_2_	*F*(000) = 584
*M**_r_* = 301.04	*D*_x_ = 2.134 Mg m^−^^3^
Monoclinic, *P*2_1_/*n*	Cu *K*α radiation, λ = 1.54184 Å
*a* = 4.95281 (3) Å	Cell parameters from 17512 reflections
*b* = 14.0515 (1) Å	θ = 4.5–78.8°
*c* = 13.5208 (1) Å	µ = 6.46 mm^−^^1^
β = 95.1372 (6)°	*T* = 100 K
*V* = 937.19 (1) Å^3^	Block, colorless
*Z* = 4	0.21 × 0.04 × 0.04 mm

### Data collection

**Table:**

XtaLAB Synergy, Dualflex, HyPix diffractometer	2038 independent reflections
Radiation source: micro-focus sealed X-ray tube, PhotonJet (Cu) X-ray Source	2010 reflections with *I* > 2σ(*I*)
Mirror monochromator	*R*_int_ = 0.030
Detector resolution: 10.0000 pixels mm^-1^	θ_max_ = 79.7°, θ_min_ = 4.6°
ω scans	*h* = −5→6
Absorption correction: gaussian (CrysAlisPro; Rigaku OD, 2023)	*k* = −17→17
*T*_min_ = 0.490, *T*_max_ = 1.000	*l* = −17→17
24751 measured reflections	

### Refinement

**Table:**

Refinement on *F*^2^	3 restraints
Least-squares matrix: full	Hydrogen site location: difference Fourier map
*R*[*F*^2^ > 2σ(*F*^2^)] = 0.028	All H-atom parameters refined
*wR*(*F*^2^) = 0.065	*w* = 1/[σ^2^(*F*_o_^2^) + (0.0212*P*)^2^ + 2.0497*P*] where *P* = (*F*_o_^2^ + 2*F*_c_^2^)/3
*S* = 1.09	(Δ/σ)_max_ < 0.001
2038 reflections	Δρ_max_ = 0.73 e Å^−^^3^
159 parameters	Δρ_min_ = −0.50 e Å^−^^3^

### Special details

**Table:**

Geometry. All esds (except the esd in the dihedral angle between two l.s. planes) are estimated using the full covariance matrix. The cell esds are taken into account individually in the estimation of esds in distances, angles and torsion angles; correlations between esds in cell parameters are only used when they are defined by crystal symmetry. An approximate (isotropic) treatment of cell esds is used for estimating esds involving l.s. planes.

### Fractional atomic coordinates and isotropic or equivalent isotropic displacement parameters (Å^2^)

**Table:**

	*x*	*y*	*z*	*U*_iso_*/*U*_eq_	Occ. (<1)
Br9	0.19184 (5)	0.35928 (2)	0.19637 (2)	0.02267 (9)	
F12	0.8845 (8)	0.5446 (3)	0.3711 (3)	0.0259 (3)	0.515 (6)
F13	0.5295 (8)	0.5812 (3)	0.4473 (3)	0.0259 (3)	0.515 (6)
F14	0.6521 (9)	0.6697 (4)	0.3275 (4)	0.0259 (3)	0.515 (6)
F12A	0.8549 (8)	0.5453 (3)	0.4018 (3)	0.0259 (3)	0.485 (6)
F14A	0.7062 (9)	0.6639 (4)	0.3163 (4)	0.0259 (3)	0.485 (6)
F13A	0.4825 (8)	0.6121 (3)	0.4364 (3)	0.0259 (3)	0.485 (6)
O2	1.2074 (4)	0.20748 (12)	0.50222 (14)	0.0254 (4)	
O10	0.2781 (4)	0.57289 (12)	0.23355 (13)	0.0249 (4)	
N1	1.0965 (4)	0.11662 (14)	0.48774 (16)	0.0220 (4)	
N3	1.0442 (4)	0.27473 (15)	0.45145 (17)	0.0254 (4)	
N6	0.7135 (5)	0.05515 (16)	0.3947 (2)	0.0298 (5)	
H6A	0.568 (7)	0.065 (2)	0.373 (3)	0.029 (9)*	
H6B	0.748 (7)	0.000 (3)	0.427 (3)	0.033 (9)*	
N7	0.6329 (4)	0.27298 (14)	0.35067 (15)	0.0194 (4)	
H7	0.525 (6)	0.241 (2)	0.316 (2)	0.024 (8)*	
C4	0.8417 (5)	0.22775 (16)	0.40856 (17)	0.0195 (4)	
C5	0.8708 (5)	0.12796 (17)	0.43003 (18)	0.0197 (4)	
C8	0.6209 (5)	0.36888 (17)	0.34691 (18)	0.0198 (5)	
H8	0.748 (6)	0.400 (2)	0.393 (2)	0.019 (7)*	
C9	0.4468 (5)	0.42186 (16)	0.28686 (17)	0.0192 (4)	
C10	0.4376 (5)	0.52457 (17)	0.28565 (17)	0.0199 (5)	
C11	0.6293 (5)	0.58284 (17)	0.36048 (18)	0.0224 (5)	

### Atomic displacement parameters (Å^2^)

**Table:**

	*U*^11^	*U*^22^	*U*^33^	*U*^12^	*U*^13^	*U*^23^
Br9	0.02440 (14)	0.01774 (14)	0.02471 (14)	−0.00003 (9)	−0.00423 (9)	−0.00055 (9)
F12	0.0292 (8)	0.0196 (6)	0.0282 (7)	−0.0003 (6)	−0.0016 (6)	−0.0005 (7)
F13	0.0292 (8)	0.0196 (6)	0.0282 (7)	−0.0003 (6)	−0.0016 (6)	−0.0005 (7)
F14	0.0292 (8)	0.0196 (6)	0.0282 (7)	−0.0003 (6)	−0.0016 (6)	−0.0005 (7)
F12A	0.0292 (8)	0.0196 (6)	0.0282 (7)	−0.0003 (6)	−0.0016 (6)	−0.0005 (7)
F14A	0.0292 (8)	0.0196 (6)	0.0282 (7)	−0.0003 (6)	−0.0016 (6)	−0.0005 (7)
F13A	0.0292 (8)	0.0196 (6)	0.0282 (7)	−0.0003 (6)	−0.0016 (6)	−0.0005 (7)
O2	0.0264 (9)	0.0158 (8)	0.0317 (9)	−0.0015 (7)	−0.0105 (7)	0.0016 (7)
O10	0.0278 (9)	0.0194 (8)	0.0261 (9)	0.0031 (7)	−0.0062 (7)	0.0032 (7)
N1	0.0236 (10)	0.0156 (9)	0.0258 (10)	0.0000 (8)	−0.0031 (8)	0.0003 (8)
N3	0.0263 (10)	0.0171 (10)	0.0308 (11)	0.0009 (8)	−0.0090 (9)	0.0023 (8)
N6	0.0219 (11)	0.0165 (10)	0.0478 (14)	−0.0013 (8)	−0.0142 (10)	0.0046 (10)
N7	0.0190 (9)	0.0147 (9)	0.0238 (10)	0.0004 (8)	−0.0024 (8)	0.0006 (8)
C4	0.0198 (11)	0.0166 (11)	0.0216 (11)	0.0012 (8)	−0.0009 (8)	0.0009 (9)
C5	0.0194 (10)	0.0174 (11)	0.0218 (11)	0.0019 (8)	−0.0005 (9)	0.0015 (9)
C8	0.0216 (11)	0.0166 (11)	0.0210 (11)	0.0009 (9)	0.0008 (9)	−0.0009 (9)
C9	0.0204 (11)	0.0166 (11)	0.0202 (11)	−0.0018 (9)	−0.0002 (8)	−0.0007 (8)
C10	0.0209 (11)	0.0179 (11)	0.0209 (11)	−0.0004 (9)	0.0006 (8)	0.0015 (9)
C11	0.0256 (12)	0.0180 (11)	0.0227 (11)	−0.0004 (9)	−0.0026 (9)	0.0029 (9)

### Geometric parameters (Å, º)

**Table:**

Br9—C9	1.894 (2)	N6—C5	1.347 (3)
F12—C11	1.369 (5)	N6—H6A	0.76 (4)
F13—C11	1.314 (4)	N6—H6B	0.89 (4)
F14—C11	1.307 (5)	N7—C8	1.350 (3)
F12A—C11	1.314 (5)	N7—C4	1.393 (3)
F14A—C11	1.356 (6)	N7—H7	0.82 (3)
F13A—C11	1.373 (5)	C4—C5	1.437 (3)
O2—N3	1.385 (3)	C8—C9	1.353 (3)
O2—N1	1.397 (3)	C8—H8	0.95 (3)
O10—C10	1.217 (3)	C9—C10	1.444 (3)
N1—C5	1.314 (3)	C10—C11	1.557 (3)
N3—C4	1.294 (3)		
			
N3—O2—N1	110.46 (17)	C8—C9—C10	125.1 (2)
C5—N1—O2	105.97 (18)	C8—C9—Br9	118.96 (18)
C4—N3—O2	105.58 (19)	C10—C9—Br9	115.99 (17)
C5—N6—H6A	120 (3)	O10—C10—C9	125.6 (2)
C5—N6—H6B	114 (2)	O10—C10—C11	114.1 (2)
H6A—N6—H6B	118 (3)	C9—C10—C11	120.1 (2)
C8—N7—C4	120.3 (2)	F14—C11—F13	111.9 (3)
C8—N7—H7	120 (2)	F12A—C11—F14A	105.3 (3)
C4—N7—H7	119 (2)	F14—C11—F12	107.1 (3)
N3—C4—N7	121.7 (2)	F13—C11—F12	108.5 (3)
N3—C4—C5	110.3 (2)	F12A—C11—F13A	106.8 (3)
N7—C4—C5	127.9 (2)	F14A—C11—F13A	105.4 (3)
N1—C5—N6	123.5 (2)	F14—C11—C10	109.6 (3)
N1—C5—C4	107.6 (2)	F13—C11—C10	108.3 (2)
N6—C5—C4	128.7 (2)	F12A—C11—C10	120.7 (3)
N7—C8—C9	126.5 (2)	F14A—C11—C10	109.5 (3)
N7—C8—H8	114.1 (18)	F12—C11—C10	111.4 (3)
C9—C8—H8	119.4 (18)	F13A—C11—C10	108.1 (2)
			
N3—O2—N1—C5	−0.3 (3)	Br9—C9—C10—O10	1.6 (3)
N1—O2—N3—C4	0.5 (3)	C8—C9—C10—C11	−2.8 (4)
O2—N3—C4—N7	179.6 (2)	Br9—C9—C10—C11	177.15 (17)
O2—N3—C4—C5	−0.5 (3)	O10—C10—C11—F14	−24.2 (4)
C8—N7—C4—N3	−7.5 (4)	C9—C10—C11—F14	159.8 (3)
C8—N7—C4—C5	172.6 (2)	O10—C10—C11—F13	98.1 (3)
O2—N1—C5—N6	176.1 (2)	C9—C10—C11—F13	−77.9 (3)
O2—N1—C5—C4	0.0 (3)	O10—C10—C11—F12A	−161.5 (3)
N3—C4—C5—N1	0.4 (3)	C9—C10—C11—F12A	22.5 (4)
N7—C4—C5—N1	−179.7 (2)	O10—C10—C11—F14A	−39.1 (4)
N3—C4—C5—N6	−175.5 (3)	C9—C10—C11—F14A	144.9 (3)
N7—C4—C5—N6	4.4 (4)	O10—C10—C11—F12	−142.7 (3)
C4—N7—C8—C9	173.0 (2)	C9—C10—C11—F12	41.4 (3)
N7—C8—C9—C10	179.2 (2)	O10—C10—C11—F13A	75.2 (3)
N7—C8—C9—Br9	−0.7 (4)	C9—C10—C11—F13A	−100.8 (3)
C8—C9—C10—O10	−178.3 (2)		

### Hydrogen-bond geometry (Å, º)

**Table:**

*D*—H···*A*	*D*—H	H···*A*	*D*···*A*	*D*—H···*A*
N6—H6*A*···O10^i^	0.77 (4)	2.14 (4)	2.871 (3)	159 (4)
N6—H6*B*···N1^ii^	0.90 (4)	2.11 (4)	2.995 (3)	169 (3)
N7—H7···Br9	0.81 (3)	2.76 (3)	3.127 (2)	109 (2)
C8—H8···F12	0.95 (3)	2.17 (3)	2.799 (5)	123 (2)
C8—H8···F12*A*	0.95 (3)	2.11 (3)	2.808 (5)	129 (2)
C8—H8···N3	0.95 (3)	2.38 (3)	2.759 (3)	103.3 (19)

Symmetry codes: (i) −*x*+1/2, *y*−1/2, −*z*+1/2; (ii) −*x*+2, −*y*, −*z*+1.
